# Socio-economic determinants of ownership and use of treated bed nets in Nigeria: results from a cross-sectional study in Cross River and Bauchi States in 2011

**DOI:** 10.1186/1475-2875-13-316

**Published:** 2014-08-13

**Authors:** Morwell Eteng, Steven Mitchell, Lawal Garba, Onebieni Ana, Mohammed Liman, Anne Cockcroft, Neil Andersson

**Affiliations:** CIET Trust, 71 Oxford Road Saxonwold, Johannesburg, 2196 South Africa; CIET/PRAM, Department of Family Medicine, McGill University, Monrteal, Canada; Community Health Department, Government of Cross River State, Cross River State, Nigeria; Bauchi State Agency for the control of HIV/AIDS, tuberculosis/leprosy and malaria (BACATMA), Bauchi, Nigeria; CIET Trust Botswana, PO Box 1240, Gaborone, Botswana

**Keywords:** Malaria, Bed nets, Nigeria, Equity, Ownership, Use

## Abstract

**Background:**

Poor people bear a disproportionate burden of malaria and prevention measures may not reach them well. A study carried out to examine the socio-economic factors associated with ownership and use of treated bed nets in Cross River and Bauchi States of Nigeria took place soon after campaigns to distribute treated bed nets.

**Methods:**

A cross-sectional household survey about childhood illnesses among mothers of children less than four years of age and focus group discussions in 90 communities in each of the two states asked about household ownership of treated bed nets and their use for children under four years old. Bivariate and multivariate analyses examined associations between socio-economic and other variables and these outcomes in each state.

**Results:**

Some 72% of 7,685 households in Cross River and 87% of 5,535 households in Bauchi State had at least one treated bed net. In Cross River, urban households were more likely to possess bed nets, as were less-poor households (enough food in the last week), those with a male head, and those from communities with a formal health facility. In Bauchi, less-poor households and those with a more educated head were more likely to possess nets. In households with nets, only about half of children under four years old always slept under a net: 54% of 11,267 in Cross River and 57% of 11,277 in Bauchi. Factors associated with use of nets for young children in Cross River were less-poor households, fewer young children in the household, more education of the father, antenatal care of the mother, and younger age of the child, while in Bauchi the factors were a mother with more education and antenatal care, and younger age of the child. Some focus groups complained of distribution difficulties, and many described misconceptions about adverse effects of nets as an important reason for not using them.

**Conclusion:**

Despite a recent campaign to distribute treated bed nets, disadvantaged households were less likely to possess them and to use them for young children. Efforts are needed to reach these households and to dispel fears about dangers of using treated nets.

## Background

Despite recent advances, malaria remains a major cause of morbidity and mortality, especially in sub-Saharan Africa, and 90% of global malaria deaths occur in sub-Saharan Africa [1]. It is recognized that poor people bear a disproportionate burden of malaria deaths [2], yet malaria control measures do not reach poor people well [3]. There are more cases and deaths from malaria in Nigeria than in any other country; malaria is responsible for 60% of outpatient visits and 30% of hospitalizations in children under five years old in Nigeria and contributes to perhaps 11% of maternal mortality [4].

In 2011, the Roll Back Malaria Partnership updated the 2008 Global Malaria Action Plan (GMAP), retaining the objective of reducing malaria cases by 75% from 2000 levels by 2015 [5]. Insecticide-treated bed nets are an important component of malaria prevention efforts. Surveys in 2000 and 2004/6 across Nigeria, Senegal, Zambia, and Uganda noted increases in bed net ownership and use as a result of efforts to increase awareness and availability of treated bed nets [6]. An indicator for measuring progress against the GMAP targets, as well as for progress against the Millenium Development Goal (MDG) 6, is the proportion of children aged under five years old who slept under a treated bed net the previous night [1].

A 2011 household survey in Cross River and Bauchi States of Nigeria provided data to allow examination of socio-economic factors related to households possessing treated bed nets, and children under four years old sleeping under treated bed nets. This paper reports on this analysis. Bauchi State, located in the north of Nigeria and within the Sahel Savannah region, is predominantly Muslim with a tradition of polygamy and extended households, while Cross River State, located in the southeast of the country in the Niger Delta/rain forest region, is predominantly Christian with typically a more nuclear household structure.

The data collection for the survey took place between July and September 2011. Coincidentally, between 2009 and 2012, the Government of Nigeria, together with development partners, undertook mass distribution campaigns intended to provide universal coverage with insecticide-treated bed nets across the country [7]. In Bauchi, the mass distribution took place between January and April 2010; it provided up to two vouchers per household for treated nets to be redeemed at distribution points [8]. In Cross River, the first phase of the ‘fill in’ distribution campaign took place in nine of the 18 local government authorities (LGAs) in the north of the state in January and February 2011, with one further LGA covered in July/August 2011, and the second phase took place in six LGAs in September to October 2011, and in the remaining two in January and February 2012. Except in the two urban LGAs, campaign workers distributed and hung nets directly in households [9].

## Methods

In 2011, a household survey on prevention and treatment of childhood illnesses formed part of a programme to support evidence-based planning of health services in two states of Nigeria [10, 11]. The stratified, last stage random, cluster sample of enumeration areas from the 2006 census comprised 90 clusters in each state (Bauchi and Cross River): ten sites in each of three focus LGAs and 60 among the remaining LGAs, to give state-level representation. The cluster in each community comprised contiguous households radiating from a random starting point, to collect data on about 100 children under four years old. There was no subsampling within the cluster. Between July and September 2011, trained fieldworkers administered a questionnaire to mothers of children aged less than four years. The questionnaire asked about the mother’s most recent pregnancy and childbirth care and outcomes, and about childhood illnesses and treatment and related issues. It asked, for each child under four years old whether that child always slept under a treated bed net in the malaria season. The field teams also administered a questionnaire to each household about demographics and socio-economic status, which included a question about possession of any treated bed nets. They interviewed key informants in each community to get information about access to health services.

Trained teams returned to the same communities in January 2012 and conducted separate male and female focus group discussions in each community. The participants for the separate male and female groups were drawn from among the households included in the household survey. Each group comprised some eight to 12 participants. The facilitators used a guide that presented the findings from the household survey about access to bed nets in each state and, based on this evidence, invited discussion about the perceived reasons for a lack of ownership and use. The trained reporters took notes during the discussions, and afterwards, together with the facilitators, prepared reports on the discussions.

### Ethical approval

The Ministry of Health in each state gave formal ethical approval for the study (Cross River – reference number CRS/MH/CSG/E-H/018/Vol.1/23, dated 23 June 2011; Bauchi - reference number MOH/ASS/166/V.1, dated 16 June, 2011). The field team leaders sought consent for the survey from leaders in each community, and interviewers sought verbal consent from the head of each household, as well as from each individual respondent. Interviewers did not record any names or identifying information and were trained not to proceed with any interview unless they could do so without being overheard.

### Analysis

Different operators entered the data twice with validation to minimize keystroke errors using Epi Info. Analysis relied on CIETmap open source software [12] that offers a user-friendly interface with the popular statistical programming language R. All estimates were weighted proportional to the population in each state, including rural and urban characteristics, and allowing for the over-sampling in the three focus LGAs in each state.

The analysis handled the findings from the two states separately. There is no intention that the two states together represent the situation in the whole of Nigeria, and the overall project under which the survey was conducted focuses on supporting evidence-based health planning at state level [10, 11]. Bivariate and then multivariate analyses examined associations between potential determinants and the outcome of interest using the Mantel Haenszel procedure [13], adjusted for clustering [14]. The multivariate analysis started with saturated models of potential determinants, and backwards elimination, based on the cluster adjusted Mantel Haenszel Chi square, continued until only variables significantly associated with the outcome remained. The odds ratio (OR) with the cluster adjusted 95% confidence interval (CI) serve to describe associations in the analyses.

A raster map of bed net coverage, created using CIETmap, combined the population relevance of each sample site with space (using inverse-distance weighted interpolation) to provide a population-weighted extension of each colour in the map legend [15].

The analysis examined associations with two outcomes: whether the household owned treated bed nets, and among households with treated nets, whether children under four years old always slept under a treated bed net during the malaria season. The equity-related variables at household level included: sex of the household head (male-headed or female-headed household), education of the household head (less than or more than junior secondary education), access to safe drinking water (‘safe’ sources including taps, bore holes with pumps and tube wells), whether the household had enough food in the previous week (as an indicator of absolute poverty), household construction (with good construction meaning zinc roof and concrete walls, as opposed to thatch/mud/timber), crowding (more than two people per room), occupation of the main breadwinner (lower or higher paying occupation), and perceived relative financial situation of the household (above or below the community average). At community level variables included: urban or rural location, electricity in the community and presence of a formal health facility in the community. The analysis considered additional factors in relation to whether young children always slept under a treated bed net: age and sex of the child, education of the parents, whether the mother had four or more antenatal visits in the last pregnancy, and number of children under three years old in the household (split between two or fewer and three or more). Due to interaction in the Bauchi model for bed net use, an additional variable combined maternal education and antenatal (ANC) visits (mother having some formal education + four or more ANC visits against all other combinations).

A secondary analysis examined factors related to ownership and use of treated bed nets, excluding those LGAs in Cross River State not covered by the distribution campaign before the household data collection.

Two of the authors conducted a thematic analysis of focus group responses on three topics: problems getting treated bed nets, why children do not sleep under nets even when the household has them, and what could convince people to use bed nets. The two investigators read through the focus group reports to identify common themes emerging for each topic, and extracted relevant quotes.

## Results

Table 1 shows the number of children, households and communities included in the study. Across the 90 communities, the number of households visited was higher in Cross River, but the number of children aged 0–47 months included in the sample was very similar between Cross River and Bauchi. Household size is typically larger in Bauchi than in Cross River.

**Table 1:** **Number of communities, households and children in the sample for both states**

	Bauchi State	Cross River State
Number of communities	90	90
Number of households	5,535	7,685
Number of children aged 0–47 months	11,277	11,267

### Ownership of bed nets

**Figure 1:**
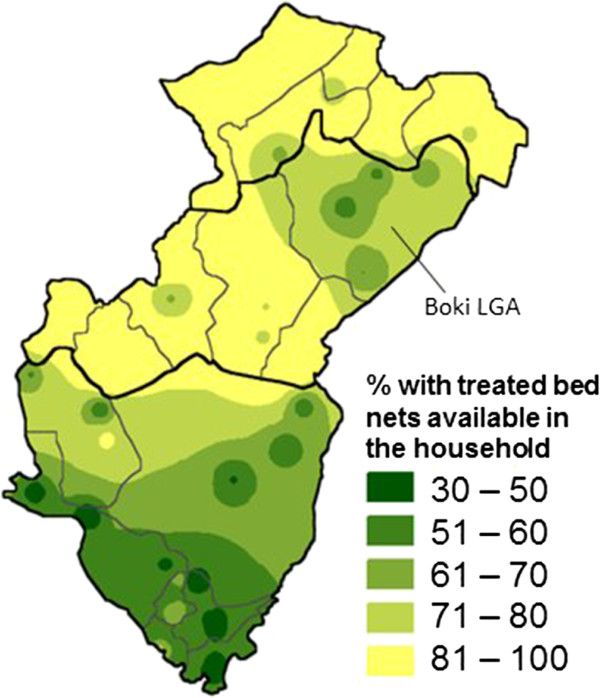
**Map showing the variation in the proportion of households in the survey owning at least one treated bed net across Cross River State.** There is a clear demarcation between the LGAs in the north, where the mass distribution campaign took place before the household data collection, and those in the south, where the campaign took place after the household data collection. The campaign in Boki LGA took place after the household data collection.

In both states a high proportion of households had a least one treated bed net (Table 2). The proportion was higher in Bauchi (87%) where the campaign to distribute bed nets was completed across the whole state before data collection, than in Cross River (72%). In Cross River, there was a marked variation across the state (Figure 1). The LGAs in the north of the state, where the campaign to distribute free treated bed nets took place before the household data collection for this study, had clearly higher coverage with bed nets than those in the south of the state, where the distribution campaign took place after the household data collection.

**Table 2:** **Availability of treated bed nets in the household and potential determinants of availability in Bauchi and Cross River States**

Factors	Weighted % (fraction)	
	Bauchi State	Cross River State
**Outcome**		
Households with treated bed nets	87 (4,853/5,523)	72 (5,718/7,664)
**Household level characteristics**		
Household head has higher than junior secondary education	27 (1,495/5,492)	63 (4,735/7,541)
Female-headed household	1 (44/5,532)	17 (1,321/7,682)
Self-perceived above average financial situation	81 (4,420/5,527)	65 (4,954/7,655)
Household had enough food in previous week	89 (4,968/5,516)	81 (6,174/7,646)
Household head has high-paying occupation	21 (1,174/5,478)	42 (3,259/7,613)
Household with safe water source	42 (2,057/5,531)	39 (3,237/7,653)
Household with good construction	20 (1,071/5,487)	66 (5,054/7,658)
Household with not more than two people per room	35 (1,865/5,511)	33 (2,572/7,668)
**Community level characteristics**		
Urban household	23 (1,081/5,535)	33 (2,566/7,685)
Households from communities with electricity	47 (2,653/5,503)	68 (5,298/7,353)
Households from communities with a formal health facility in the community	62 (3,503/5,532)	77 (5,877/7,519)

Table 2 also shows the frequencies of household and community-level factors potentially related to bed net ownership, with some differences apparent between the two states. Most of the households reported they had enough food in the last week, the majority were rural and some two-thirds or more were from communities that included a formal health facility.

Table 3 shows the final multivariate models of factors related to bed net ownership (having at least one treated bed net in the household). In both states, less-poor households (with enough food in the previous week) were more likely to own a treated bed net than very poor households (without enough food). In Cross River, households from rural areas and female-headed households were less likely to possess nets, while those from communities with a formal health facility were more likely to possess nets. In Bauchi, households with a more educated head were more likely to possess nets. The secondary analysis excluding the LGAs in Cross River not covered by the distribution campaign prior to household data collection found associations similar to those of the main analysis.

**Table 3:** Factors related to bed net ownership*

Variables	OR	OR a	95% CI ca for OR a
**Cross River**			
Female-headed household	0.71	0.72	0.63-0.82
Households with enough food in the previous week	1.78	1.76	1.45-2.13
Urban household	0.62	0.61	0.42-0.90
Households from communities with a formal health facility in the community	1.91	1.91	1.20-3.06
**Bauchi**			
Household head has junior secondary or higher education	1.43	1.39	1.01-1.92
Households with enough food in the previous week	1.75	1.69	1.20-2.37

*Initial models included household head education, household head sex, self-perceived financial situation, food security, household head occupation, safety of water source, household construction, household crowding, urban/rural setting, electricity in household, and whether or not there was a formal health facility in the community.

### Views from the focus groups

Some focus group participants in Cross River State complained that not enough bed nets were available through the distribution campaign and voiced suspicions that the distributors diverted the supplied nets.

*“Some health workers keep the nets that are supposed to be for distribution.”* (Male group, Cross River)

*“The people in charge of the bed nets will sometimes give nets to only five persons and then say the nets are finished but will later take the remaining nets to their houses and sell them.”* (Female group, Cross River)

In Bauchi, some focus group participants complained that they did not receive enough nets for their family size.

*“I have more than six children with my wives but I was only given two nets.”* (Male group, Bauchi)

Some noted problems with the vouchers needed for getting nets.

*“I have no card so I was not given the bed net.”* (Female group, Bauchi)

Other groups suggested the distribution system worked well.

*“All women were gathered at Jauro’s house (community leader) and collected the nets after presenting the card that was issued to every woman in this community.”* (Female group, Bauchi)

### Use of treated bed nets

Among households that owned at least one treated bed net, only about half of children aged 0–47 months in Cross River and Bauchi always slept under a treated bed net in the malaria season (Table 4). Considering *all* households, some 51% (5,786/11,112) of children aged 0–47 months in Bauchi and 41% (4,822/11,159) in Cross River always slept under a treated net in the malaria season. Table 4 also shows the frequencies of factors potentially related to use of treated bed nets among children aged 0–47 months in households owning nets*.* Again, there were some differences apparent between the two states, with higher numbers of young children (under three years old) in the households in Bauchi.

**Table 4:** **Consistent use of treated bed for children aged 0–47 months (in households with a least one treated net) and potential determinants of use**

Factors	Weighted % (fraction)	
	Bauchi State	Cross River State
**Outcome**		
Child always slept under a treated bed net	57 (5,666/9,757)	54 (4,662/8,366)
**Child level characteristics**		
Female child	50 (4,946/9,878)	51 (4,300/8,412)
Child less than two years of age	52 (5,178/9,879)	51 (4,329/8,412)
Mother has junior secondary education or higher	-	65 (5,251/8,332)
Mother has some formal education	20 (2,052/9,867)	-
Father has junior secondary education or higher	26 (2,558/9,779)	72 (5,736/8,054)
Mother received four or more ANC visits	46 (3,937/8,665)	44 (3,612/6,414)
**Household level characteristics**		
Child from a household with enough food in the previous week	91 (9,022/9,849)	83 (6,933/8,374)
Child from a household with self-perceived average or above financial situation	83 (8,021/9,869)	67 (5,533/8,387)
Child from a household where household head has higher paying occupation	22 (2,104/9,786)	43 (3,591/8,337)
Child from female-headed households	1 (49/9,874)	15 (1,255/8,408)
Child from a household with less than three children under three years in the household	55 (5,310/9,879)	82 (6,914/8,413)
**Community level characteristics**		
Child from urban community	19 (1,659/9,879)	31 (2,537/8,413)
Child from community with a formal health facility in the community	61 (6,237/9,879)	80 (6,631/8,264)

Table 5 shows the final multivariate models of factors related to bed net use among children aged 0–47 months in households possessing at least one treated bed net. In both states, a child less than two years old was more likely to always sleep under a treated bed net than a child aged two years or older. Children whose mothers had attended for at least four ANC visits in their last pregnancy were more likely to sleep under a bed net; in Bauchi, this was only a factor when the mother had some formal education. In Cross River, a child from a household with less than two children under three years of age was more likely to always sleep under a bed net. The analysis excluding LGAs in Cross River that had not been covered by the distribution campaign found similar associations with use of bed nets to those described by the main analysis.

**Table 5:** Factors related to bed net use among children aged 0–47 months among those who have bed nets available in the household*

Cross River	OR	OR a	95% CI ca for OR a
Child whose father had junior secondary or higher education	1.37	1.26	1.07-1.49
Child less than two years of age	1.63	1.58	1.43-1.75
Child whose mother had four or more ANC visits	1.33	1.27	1.08-1.49
Child from a household with enough food in the previous week	1.32	1.24	1.02-1.51
Child from a household with two or less children under three years in the household	1.57	1.47	1.21-1.79
**Bauchi**	**OR**	**OR a**	**95% CI ca for OR a**
Child whose mother had some formal education and received four or more ANC visits	1.72	1.71	1.38-2.12
Child less than two years of age	1.10	1.09	1.01-1.17

*Initial models included sex of child, age of child, maternal education, paternal education, ANC visits, household food security, self-perceived financial situation, household head occupation, sex of household head, household crowding, rural/urban setting, and whether or not there was a formal health facility in the community.

### Views from the focus groups

Focus group respondents in both states said that poor ventilation and overcrowding of small rooms inhibits the use of treated bed nets. Some participants explained that they did not have enough nets to cover all their young children.

*“We have many children but only one net. This is why few children get to sleep inside one.”* (Female group, Bauchi)

Many focus groups cited concerns and beliefs about adverse effects of treated bed nets as their reason for not using them. They mentioned itching, nausea, and even infertility or death.

*“In one of our neighbouring communities, someone put the net outside in order to air it. His cow ate the net and fell down dead soon afterwards.”* (Male group, Bauchi)

*“Some believe that since the net can kill a mosquito it can kill a human being too.”* (Female group, Bauchi)

## Discussion

A high proportion of households in both states (87% in Bauchi and 72% in Cross River) had at least one treated bed net. The higher proportion possessing bed nets in Bauchi probably reflects the timing of the data collection in relation to the campaigns for universal distribution of free treated bed nets. In Bauchi, the data collection took place after completion of the distribution campaign across the whole state, while in Cross River it took place after the first phase of the distribution campaign (that covered ten of the 18 LGAs) and before the second phase (that covered the remaining eight LGAs). The coverage with treated bed nets in this survey is much higher than that reported in the 2008 Demographic and Health Survey (DHS), which took place before the mass distribution campaigns: 7.4% in Bauchi and 15.7% in Cross River [16]. Coverage figures from this survey are similar to the coverage figures reported by the Multiple Indicator Cluster Survey (MICS) which collected data in February and March 2011: Bauchi 82% and Cross River 67% [17].

Despite the high coverage with bed nets in areas covered by the distribution campaign, in both states the more disadvantaged households were less likely to have a treated bed net. The present survey was not designed to assess the effectiveness of the campaign in reaching poor households, and this was not a study measuring the situation before and after the distribution campaign. However, since the most disadvantaged households had lower ownership of treated nets *even after* the campaign, this suggests that the campaign to distribute free nets may not have reached the most needy households effectively. Difficulty in accessing the places where bed nets were distributed may have been an issue for some households, especially in Bauchi. Although most of the distribution in Cross River was house-to-house, nevertheless households in Cross River were more likely to have a net if there was a health facility in their community. A national survey in Nigeria in 2005, prior to the mass distribution campaigns, found that more educated households and those in communities with a health facility were more likely to possess a treated bed net [18]. A national survey among 2,348 pregnant women in Nigeria conducted in 2008 found that women knowing about the role of bed nets for malaria prevention were more likely to possess nets, but did not find an association with overall educational level [19].

Both states in the present study had relatively low utilization of bed nets among children aged 0–47 months in households owning nets. These figures for use of treated bed nets for children can be compared with those from the 2011 MICS survey. While the present study found among all households that 51% of children aged 0–47 months in Bauchi and 41% in Cross River always slept under a net in the malaria season, the MICS survey found 13% of children under five years old in Bauchi and 41% in Cross River slept under a net the previous night [17]. The low figure from the MICS survey in Bauchi is likely to be because the survey was undertaken in the dry season in that region of the country. Other studies in Nigeria have also reported low levels of use of treated bed nets even when the household possesses them [20, 21].

The present study found children were more likely to sleep under treated bed nets if their parents were more educated and if their mothers had attended ANC. This may be because ANC visits are a source of information about the importance of bed nets. Additionally, educated parents may be better able to appreciate the importance of treated nets in malaria prevention and to understand the information included in the public awareness campaigns.

One reason for not using treated bed nets for young children may be that the household does not have enough nets. The survey questionnaire did not ask about number of treated nets owned by the household. However, the finding in Cross River that a child with fewer young siblings was more likely to sleep under a net supports the idea that shortage of nets could be an issue in larger households. Focus groups also frequently complained about not receiving enough nets for the needs of their household. When they have insufficient nets for all their young children, parents may choose to use nets particularly for very young children, as suggested by the finding that children under two years old were more likely to sleep under nets than older children.

Apart from availability issues, there were prevailing beliefs and misconceptions that inhibit the use of treated bed nets. Many focus group participants expressed concerns about adverse effects of treated bed nets. Some of the concerns, such as the nets being hot and uncomfortable, may have a factual basis. Many households have small, poorly ventilated sleeping areas where nets may indeed exacerbate uncomfortable conditions. However, many concerns were based on misconceptions about the health risks of using bed nets.

### Limitations

These analyses are based on a cross-sectional survey and thus can only report on associations rather than draw conclusions about causality. As the study was carried out as part of a larger investigation about prevention and management of childhood illnesses, the information collected about economic status was relatively limited; nevertheless associations with ownership and use of bed nets were apparent. Importantly, this study was not designed to assess the effectiveness of the government campaigns to distribute treated bed nets; it did not comprise a before and after survey and it did not set out to compare areas that had been covered by the campaign with those that had not. The timing in relation to the campaigns was coincidental. It was fortuitous that the data collection took place soon after a distribution campaign in most areas. The analysis showed that disadvantaged households were less likely to possess bed nets, even after a recent mass distribution campaign.

## Conclusion

Coverage with treated bed nets in Cross River and Bauchi states was high following the recent distribution campaign, but this study provides evidence that the most disadvantaged households were less likely to possess treated bed nets, even after the campaign. Actual use of bed nets for young children lags well behind the possession of nets. Use could be improved by ensuring households receive enough nets for all their children. Many fears and misconceptions about treated bed nets persist and will need to be tackled in order to increase the use of bed nets and reach the 2015 malaria prevention targets.

## Abbreviations

ANC: Antenatal care
CI: Confidence interval
DFATD: Foreign Affairs, Trade and Development Canada
IDRC: International Development Research Centre
LGA: Local Government Authority
NEHSI: Nigeria Evidence-based health Services Initiative
OR: Odds ratio
RBM: Roll Back Malaria
TB: Tuberculosis
WHO: World Health Organization.
